# Effectiveness and Clinical Value of Laparoscopic Cholecystectomy and Cholangiography in the Diagnosis of Biliary Calculi

**DOI:** 10.3389/fsurg.2022.880266

**Published:** 2022-04-27

**Authors:** Yunqing Zhao, Hongbo Tao, Yanqin Liu, Gen Sha, Xianyun Yi, Qin Qin, Dong Jin, Chengjie He, Xianghong Wu, Qing Zhou

**Affiliations:** ^1^Department of General Surgery, Wuhan Wudong Hospital (Wuhan Second Psychiatric Hospital), Wuhan, China; ^2^Department of Anesthesiology, Wuhan Wudong Hospital (Wuhan Second Psychiatric Hospital), Wuhan, China; ^3^Operating Room, Wuhan Wudong Hospital (Wuhan Second Psychiatric Hospital), Wuhan, China

**Keywords:** laparoscopic cholecystectomy, cholangiography, bile duct stones, diagnosis, effectiveness, clinical value

## Abstract

**Objective:**

To investigate the effectiveness and clinical value of cholangiography in the diagnosis of bile duct stones in laparoscopic cholecystectomy.

**Methods:**

200 patients who underwent laparoscopic cholecystectomy in our hospital from January 2017 to January 2019 were randomly divided into research group and control group, with 100 cases in each group. The research group underwent choledochotomy and exploration with the help of choledochoscope, while the control group underwent cholangiography to diagnose bile duct stones. The cure rate, residual stone rate, complication rate, intraoperative bleeding, hospital stay and patient satisfaction were compared between the two groups.

**Results:**

in the control group, 9 cases were converted to laparotomy, 20 cases of common bile duct stones, 10 cases of bile duct injury and 6 cases of common bile duct variation. In the research group, there were 2 cases of conversion to laparotomy, 12 cases of common bile duct stones, 2 cases of bile duct injury and 4 cases of common bile duct variation. The cure rate of the researchgroup was higher than that of the control group, There was significant difference between the two groups (*P* < 0.05). The residual amount of stones in the research group was lower than that in the control group, and there was significant difference between the two groups (*P* < 0.05). The incidence of postoperative complications in the research group was lower than that in the control group, and there was significant difference between the two groups (*P* < 0.05). The patient satisfaction in the research group was higher than that in the control group, and there was significant difference between the two groups (*P* < 0.05). The intraoperative blood output of the research group was lower than that of the observation group, and there was significant difference between the two groups (*P* < 0.05).

**Conclusion:**

cholangiography is an effective method for the diagnosis of bile duct stones in laparoscopic cholecystectomy. Clarifying the variation and anatomical structure of bile duct is helpful to improve the surgical cure rate, reduce the residual rate of postoperative stones and the incidence of complications, reduce the amount of intraoperative bleeding, shorten the hospital stay, and promote the postoperative rehabilitation of patients.

## Introduction

Benign gallbladder disease is a common disease in general surgery. It mainly includes gallstones, polypoid lesions of gallbladder, cholecystitis, etc. The incidence rate showed an increasing trend year by year. Common symptoms of gallbladder diseases include severe pain in the right upper abdomen, abdominal distension, loss of appetite, yellow urine, yellow skin, etc. Different causes of gallbladder diseases will show different symptoms. For example, cholecystitis and cholecystolithiasis will cause severe pain in the right upper abdomen due to local inflammatory stimulation of the gallbladder, accompanied by decreased appetite. If the inflammation is serious, it can also be accompanied by fever and other manifestations.

Clinically, laparoscopic cholecystectomy has become the first choice for the treatment of benign gallbladder diseases (1). However, in laparoscopic cholecystectomy, choledochoscopy is often used to explore choledocholithiasis. However, due to the complex condition of some cases and the limited technical level of operators, the incidence of complications during resection is also high. The common complications include intraoperative bile duct injury and residual stones of common bile duct. Common bile duct injury is one of the most serious complications of laparoscopic cholecystectomy, with an incidence of 0.3–0.7% (2).

Therefore, how to reduce the residual rate of stones, avoid bile duct injury and prevent negative exploration of common bile duct during operation is the key issue concerned by clinicians (3, 4). Studies have shown that intraoperative cholangiography can effectively avoid unnecessary common bile duct exploration, find residual bile duct stones in time, reduce bile duct injury, and clarify bile duct lesions and anatomical abnormalities. It can effectively diagnose biliary calculi, so as to improve the surgical efficacy and prognosis of patients (2, 5, 6). This article discusses the effectiveness and clinical value of cholangiography in the diagnosis of biliary calculi during laparoscopic cholecystectomy.

## Information and Methods

### General Information

A total of 200 patients who underwent laparoscopic cholecystectomy in our hospital from January 2017 to January 2019 were selected and this study was approved by the hospital ethics committee. They were randomly divided into control group and research group, with 100 cases in each group. In the control group, there were 47 males and 53 females, aged 27–78 years, with a mean age of 52.6 ± 3.7 years. In the research group, there were 41 males and 59 females, aged 26–79 years, with a mean age of 52.8 ± 4.5 years. There was no significant difference in general data between the two groups. The two groups were comparable (see Table 1).

**Table 1:** Comparison of two groups of general data.

**Groups**	**Number of cases**	**Gender (male/female, *n*)**	**Age (year)**	**Course of disease (year)**	**Leukocyte count (×10^**9**^/L)**
control group	100	43/53	52.6 ± 3.7	3.58 ± 1.05	14.25 ± 2.98
research group	100	41/59	52.8 ± 4.5	3.64 ± 0.98	14.05 ± 3.12
χ^2^/*t*	-	0.086	0.912	0.403	0.467
*P*	-	0.768	0.316	0.688	0.639

Inclusion criteria: (1) previous history of jaundice and pancreatitis; (2) CT (computed tomography) and ultrasound in emergency patients unable to undergo magnetic resonance cholangiopancreatography (MRCP) or in patients contraindicated for MRCP suggesting a common bile duct diameter >1 cm and no common bile duct stones were found; (3) preoperative diagnosis of cystic duct stone, turbid bile in the bile duct, dilated cystic duct; (4) common bile duct stone could not be clearly exclude through preoperative CT, color ultrasound or MRCP. Exclusion criteria: (1) the presence of contraindication to the procedure; (2) allergy to contrast media; (3) lack of clinical data.

### Methods

Preoperative examinations such as MRCP, CT and ultrasound were performed in all patients. All patients underwent laparoscopic cholecystectomy with tracheal intubation under general anesthesia, and the surgical operation was completed by four-hole puncture method. In both groups, the gallbladder triangle was finely dissected and the common bile duct, cystic duct and cystic artery were isolated. In the research group, choledochotomy was performed with the help of choledochoscope, while in the control group, intraoperative cholangiography was taken to diagnose bile duct stones.

Surgical approach: (1) Control group: laparoscopic 4-hole approach, transcystic bile duct choledochotomy exploration or direct choledochotomy exploration was used. The main subxiphoid operating hole should be incised longitudinally as far up as possible, and the right margin of the hepatoduodenal ligament was incised to separate the cystic duct and the cholecystic artery. After clamping the cystic artery, the cystic artery was coagulated with the distal end of the biological clip, and the cystic duct was not cut for the time being after clamping the cystic duct. The junction of the cystic duct and common bile duct was incised longitudinally, or the common bile duct was incised directly longitudinally, the bile in the common bile duct was aspirated, and all stones were removed. Then, a choledochoscope was placed through the subxiphoid Trocar and the common bile duct was explored with the choledochoscope. The order of choledochoscopy was to explore the lower part of the common bile duct first and then the common hepatic duct. The common bile duct was examined at least three times in the above sequence to prevent residual stones in the common bile duct that could cause postoperative residual bile duct stones and bile leakage. A 20–22 gauge T-tube was placed in the common bile duct, and the T-tube was extracted and fixed from the subcostal puncture of the right midclavicular line. The common bile duct incision was interrupted suture with 5 ~ 0 or 4 ~ 0 absorbable sutures for 6 ~ 8 stitches in full layer, the gallbladder was removed, the abdominal cavity was washed, a drainage tube was placed at the Winsdow foramen, and extracted and fixed from the right anterior axillary line puncture.

Research group: the distal gallbladder neck was first clamped with a vascular clip. A small incision was made at the proximal end of the cystic duct at a distance of 1 cm from the common bile duct, a thin catheter was placed and entered at least 1 cm into the common bile duct, the angiographic tube was fixed, the open part of the cystic duct was clamped, saline was injected, and after determining whether there was any exudation, 20 mL of 30% meglumine diatrizoatewas was injected, and the injection rate should be first fast and then slow. The patient was instructed to take a supine position with the OT operating table tilted and the head down. Keep the patient's head low and feet about 15 degrees higher to ensure that the lower end of the common bile duct and the left and right hepatic ducts can be observed, and avoid retrograde biliary tract infection and acute spasm of the biliary sphincter. After the angiography, remove the angiography tube, clamp the gallbladder tube 0.5 cm away from the common bile duct with a vascular clamp, and remove the excess gallbladder tube.

When patients with Mirizzi syndrome type II~IV, intraoperative adhesion and separation of gallbladder and transverse colon, resulting in biliary intestinal fistula and adhesion of gallbladder triangle, they should be converted to laparotomy in time to avoid serious complications. The conversion to laparotomy was performed by right costal margin oblique incision and anterograde or retrograde resection.

### Observation Indicators

The surgical cure rate, residual stone rate, incidence of complications (common complications including abdominal infection, acute cholangitis, bleeding and pancreatitis) and length of hospital stay were observed, and the postoperative satisfaction of patients was investigated.

### Statistical Analysis

Select SPSS 26 0 software for data analysis, the measurement data is described by $\bar{x}\pm$ s, and the selected components are compared by *t*-test. The counting data were described by % and selected χ^2^ test for comparison between two groups. *P* < 0.05 indicates that there is a statistical difference between the two groups.

## Results

### Comparison of Preoperative Examination Results Between the Two Groups

In the control group, 62 cases underwent CT examination before operation, of which 8 cases showed unclear or mild dilation of the lower end of the common bile duct; 26 cases were examined by B-ultrasound before operation. The results showed that the lower end of common bile duct was unclear or slightly dilated in 6 cases; MRCP was performed in 12 cases before operation. The results showed that 6 cases were space occupying lesions or suspicious stones at the lower end of the common bile duct, 6 cases had tight adhesion of the gallbladder triangle and cystic duct stenosis. Among the patients who underwent intraoperative cholangiography in the research group, 34 cases underwent CT examination before operation, and 4 cases had unclear display and mild expansion of the lower segment of the common bile duct; Among 54 patients who underwent B-ultrasound examination before operation, 4 cases showed unclear display of the lower end of common bile duct; Among the 12 patients who underwent MRCP before operation, 4 cases were space occupying lesions or suspicious stones at the lower end of the common bile duct, and 4 cases had gallbladder duct variation or stenosis.

### Comparison of Intraoperative Conditions Between the Two Groups

In the research group, 2 cases were converted to laparotomy, 12 cases of bile duct stones, 2 cases of bile duct injury and 4 cases of common bile duct variation were found. In the control group, 9 cases were converted to laparotomy, 20 cases of bile duct stones, 10 cases of bile duct injury and 6 cases of common bile duct variation were found (see Table 2).

**Table 2:** Comparison of intraoperative conditions between the two groups (%).

**Groups**	**Number of cases**	**Conversion to laparotomy**	**Common bile duct stone**	**Biliary tract injury**	**Common bile duct variant**
control group	100	9 (9%)	20 (20%)	10 (10%)	6 (6%)
research group	100	2 (2%)	12 (12%)	2 (2%)	4 (4%)

### Comparison of Postoperative Related Indexes

The surgical cure rate of the research group (100%) was higher than that of the control group (82%), and there was significant difference between the two groups (*P* < 0.05). The stone residue rate of the research group (5%) was lower than that of the control group (17%), and there was significant difference between the two groups (*P* < 0.05). The incidence of complications in the research group (2 cases) was lower than that in the observation group (9 cases), and there was significant difference between the two groups (*P* < 0.05) (67.48 ± 6.18ml) the mean amount of intraoperative bleeding was lower than that in the observation group (86.63 ± 7.59 ml), and there was significant difference between the two groups (*P* < 0.05). The length of hospital stay in the research group (7.4 ± 2.6 d) was lower than that in the observation group (9.0 ± 1.3 d), and there was significant difference between the two groups (*P* < 0.05). See Table 3 for details.

**Table 3:** Cure rate and residual stone rate in both groups [n (%), $\bar{x}\pm$ s].

**Groups**	**Number of cases**	**Surgical cure rate**	**Residual stone rate**	**Complication**	**Satisfaction**	**Intraoperative bleeding (mL)**	**Length of stay in hospital (d)**
Control group	100	82 (82%)	17 (17%)	9 (9%)	87 (87%)	86.63 ± 7.59	9.0 ± 1.3
Research group	100	100 (100%)	5 (5%)	2 (2%)	98 (98%)	67.48 ± 6.18	7.4 ± 2.6
χ^2^/*t*	-	19.870	7.354	4.714	8.721	5.746	17.872
*P*	-	<0.001	0.007	0.030	0.003	0.021	<0.001

## Discussion

In Europe and the Americas, 150–200 per 100,000 people undergo cholecystectomy each year, more than 80% of which are laparoscopic procedures (7, 8). In China, with the improvement of living standards, the incidence of benign gallbladder disease is increasing year by year, and the number of patients undergoing laparoscopic cholecystectomy is also increasing every year. It has been found that laparoscopic cholecystectomy is prone to common bile duct stone omission and can cause imperceptible bile duct injury. The main reason for this is that laparoscopic cholecystectomy is performed by placing instruments in the abdominal cavity, which does not allow for palpation of the common bile duct in open surgery and can lead to unnecessary negative exploration of the common bile duct or leftover common bile duct stones in some patients (9). Early detection of common bile duct stones is significant for diagnosis and treatment. Moreover, stones located in the neck of the gallbladder in patients with chronic cholecystitis can develop secondary biliary stones in just a few days during acute attacks due to pressure changes and inflammatory irritation, leading to greater damage. Imaging is the primary method of diagnosing benign gallbladder disease. There are many types of preoperative imaging examinations, such as ultrasound, CT, ERCP (endoscopic retrograde cholangiopancreatography) and MRCP, all of which have certain application value, but each method also has certain limitations. Due to the fine anatomical structure of the intraoperative gallbladder triangle, the imaging means should be used reasonably with the surgical operation according to the actual situation, and the anatomy of various bile duct structures should be handled flexibly, which can prevent and reduce bile duct injury and bleeding effectively (10).

In recent years, it has been found that the performing transcholangiography during laparoscopic cholecystectomy can effectively clarify the presence and location of common bile duct stones, avoid unnecessary negative exploration, prevent stone residuals, detect bile duct anatomical variants and medically induced injuries in a timely manner, and improve the quality of surgery effectively (11). In this study, in laparoscopic cholecystectomy, the control group was selected to perform transcystic ductography, and of the 36 cases with successful imaging, 9 cases were intraoperatively converted to laparotomy, there were 12 cases of common bile duct stones, 2 cases of biliary tract injury, and 4 cases of common bile duct variants. Compared with the research group in which choledochotomy was performed with the aid of choledochoscopy, the probability of intraoperative conversion to laparotomy and the rate of residual stones were significantly lower, and the cure rate of the procedure and postoperative patient satisfaction were significantly higher (*P* < 0.05). The implementation of intraoperative cholangiography not only reduces biliary injury but also avoids biliary exploration, which has the advantages of reliability, effectiveness, safety, and ease of operation (12). In addition, intraoperative cholangiography can detect bile duct injury timely, distinguish the location of the distal common hepatic duct, proximal common bile duct and proximal cystic duct clearly, and help to separate the gallbladder triangle accurately, stop bleeding safely and prevent bile duct injury (13). Therefore, it is seen that cholangiography during laparoscopic cholecystectomy has positive value and significance in diagnosing biliary stones.

Intraoperative cholangiography, in the treatment of benign gallbladder disease, is considered to be beneficial in describing the biliary anatomy and identifying stones in the gallbladder. However, a part of the scholars argued that this additional imaging of the procedure brings minimal benefits and complicates the surgical conditions. Subsequently, some scholars have analyzed intraoperative cholangiography, and the results showed that this method can significantly reduce the degree of injury, thereby reducing the incidence of complications of surgery (14). The most common major complication in cholecystectomy is bile duct injury, which has a published incidence of up to 1.4%, although an incidence of 0.15–0.6% is found in literature reports, which is ~1 per 200 operations (15). A related study (16) reported that routine use of intraoperative cholangiography reduced the incidence rate of bile duct injury from 85 to 45%. Ludwig et al. (17) also showed that intraoperative cholangiography reduced the incidence rate of bile duct injury from 90 to 45%, similar to the former study. Although there are different opinions on intraoperative cholangiography, numerous studies have shown that this method reduces the incidence of surgical complications and identifies stones rapidly at the time of surgery (18).

In conclusion, in laparoscopic cholecystectomy, cholangiography is more effective in the diagnosis of biliary calculi. It can clarify the variation and anatomical structure of bile duct, avoid residual bile duct stones, reduce bile duct injury, and has high safety, which is conducive to the postoperative recovery of patients. Therefore, it is worthy of application and promotion.

## Data Availability Statement

The original contributions presented in the study are included in the article/supplementary material, further inquiries can be directed to the corresponding authors.

## Ethics Statement

The studies involving human participants were reviewed and approved by Ethics Committee of Wuhan Wudong Hospital. The patients/participants provided their written informed consent to participate in this study.

## Author Contributions

YZ, HT, YL, and GS: conception and design of the research. YZ, YL, XY, and QQ: acquisition of data. HT, GS, DJ, and CH: analysis and interpretation of the data. YZ, HT, YL, GS, XW, and QZ: statistical analysis. YZ and HT: writing of the manuscript. YL and GS: critical revision of the manuscript for intellectual content. All authors contributed to the article and approved the submitted version.

## Funding

Scientific research fund of Wuhan Municipal Health Commission, Project Number: WX20Z36.

## Conflict of Interest

The authors declare that the research was conducted in the absence of any commercial or financial relationships that could be construed as a potential conflict of interest.

## Publisher's Note

All claims expressed in this article are solely those of the authors and do not necessarily represent those of their affiliated organizations, or those of the publisher, the editors and the reviewers. Any product that may be evaluated in this article, or claim that may be made by its manufacturer, is not guaranteed or endorsed by the publisher.
